# Photo-Cross-Linked Pluronic F127 Hydrogels for Controlled Protein Delivery

**DOI:** 10.1021/acsomega.5c10776

**Published:** 2026-04-29

**Authors:** Fernando Carrascosa, Ignacio Gracia, María Jesús Ramos, Jesús Manuel García-Vargas, Juan Francisco Rodríguez, María Teresa García

**Affiliations:** Department of Chemical Engineering, 16733 University of Castilla-La Mancha, Facultad de Ciencias y Tecnologías Químicas, Avda., Camilo José Cela 12, 13071 Ciudad Real, Spain

## Abstract

Hydrogels are promising platforms for controlled delivery of biomacromolecules due to their biocompatibility, water retention, and similarity to the extracellular matrix, which supports cell interaction and tissue integration. This study presents the development and characterization of diacrylated Pluronic F127-based hydrogels for biomedical applications, focusing on controlled protein release. Bovine serum albumin (BSA) was used as a model protein to assess release behavior under varying physiological conditions. The hydrogels were synthesized via photopolymerization, and their structural integrity, swelling capacity, and stability were analyzed. Release assays under different pH levels and BSA concentrations showed that both factors significantly affected the release profile. Depending on these conditions, the release followed either a first-order or biphasic pattern, indicating both diffusion- and matrix-driven mechanisms. This tunability highlights the system’s adaptability for specific therapeutic needs requiring precise temporal protein delivery. Physicochemical analyses and kinetic modeling confirmed that release behavior can be tailored through environmental and formulation parameters. These findings demonstrate the potential of Pluronic F127 hydrogels as versatile, customizable platforms for localized therapeutic protein delivery, with broad implications in regenerative medicine, targeted drug delivery, and other biomedical fields.

## Graphical abstract

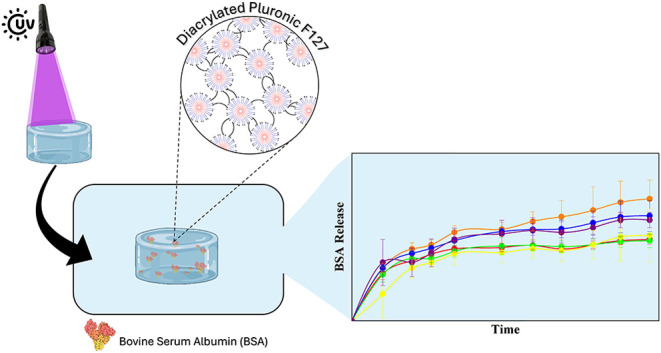

## 1 Introduction

The role of hydrogels in tissue engineering (TE) has grown significantly in recent times. These versatile materials are composed of a three-dimensional network of polymer chains and are able to hold a large amount of water while maintaining their structure.^1^ TE is generally credited with having three fundamental pillars: scaffolds, selected cells, and growth factors. Hydrogels hold great promise as effective scaffolds, providing the physical support necessary for cells to adhere, proliferate, and organize into functional tissues. Their unique ability to mimic the natural extracellular matrix or act as structural support makes them highly suitable for tissue engineering applications, and they also serve as effective drug-releasing systems.^2–7^ In comparison to traditional scaffolding materials such as metals or ceramics, hydrogels offer a more flexible and tunable platform that can be tailored to specific tissue types and degradation rates.^8–11^ Furthermore, hydrogels facilitate the diffusion of nutrients and oxygen, thereby improving cell survival and function.^12–15^

Pluronic polymers are a family of triblock copolymers containing poly­(propylene oxide) (PPO) and poly­(ethylene oxide) (PEO) arranged in the PEO–PPO-PEO manner. The most studied copolymer of this family is Pluronic F127, which exhibits a PEO/PPO ratio of 7/3 wt. Its amphiphilic nature allows for self-assembly into micelles in aqueous solution at concentrations higher than the critical micellar concentration (CMC).^16^ Pluronic F127 is thermosensitive, biodegradable, nontoxic, and biocompatible.^17,18^ The unique thermosensitivity of Pluronic F-127 hydrogels allows the formation of gels at specific temperatures, which facilitates their handling and application in biomedical systems. In fact, Pluronic F127 has been studied in resensitization of drug-resistant cancers,^19–21^ tissue engineering,^22–25^ gene carriers,^26–28^ wound healing,^29,30^ and medical diagnostics.^31^ One of the most prominent applications of Pluronic F-127 hydrogels is their use in controlled drug delivery systems.^32–35^ Because of their high-water retention capacity in the polymeric network, they are suitable materials for introducing or encapsulating drugs or biologically active molecules.

Bovine Serum Albumin (BSA) has become a valuable protein in the study and design of drug delivery systems. On one hand, this material has garnered increasing attention due to its wide availability, capacity to form complexes with a variety of pharmaceutical agents, biocompatibility, biodegradability, and notable thermal stability.^36^ More recently, it has also been associated with enhanced wound healing and tissue repair, effects that appear to be mediated through its intrinsic antioxidant activity and its ability to modulate inflammatory responses.^37,38^ Given these attributes, BSA stands out as a particularly suitable model for the initial investigation of controlled drug delivery systems, offering a reliable and well-characterized platform for studying release profiles.

Previous studies have shown that while Pluronic F127 and PEG-based hydrogels are biocompatible and have adjustable mechanical properties, they have limitations in controlled protein delivery. For Pluronic F127 systems, fast micellar dissociation and limited structural stability under physiological conditions can lead to early gel breakdown and sudden release.^39^ Likewise, protein release from PEG-based hydrogels mainly depends on network mesh size and degradation rates. This can limit long-term control and needs careful management of cross-linking density to prevent either quick depletion or very slow diffusion.^40^ These issues emphasize the need for different network designs that allow better control of protein release patterns in various environmental conditions.

In this study, Pluronic F127 was functionalized with acrylate groups. The terminal π-electron functionalization of Pluronic chains provides the hydrogel with the ability to chemically cross-link via a photoinitiated radical mechanism, thereby endowing it with a nonthermosensitive induced behavior and enhancing structural stability and strength. This functionalization strategy has been previously reported, where the terminal modification of Pluronic F127 with acrylate groups (F127DA), as well as with other reactive moieties, is of particular interest in wound healing and repair applications, even for other hydrogel precursors such as PEG–DA.^17^ The photo-cross-linkable nature of diacrylated F127 (F127DA) makes them especially attractive for encapsulation systems,^41^ the fabrication of biomedical nanoparticles,^42^ and as hydrogel platform for tissue engineering applications.^43–45^ In addition, it allows drugs or proteins into the hydrogel, as they are retained in the aqueous matrix once cross-linked, in an efficient and safe process. Bovine serum albumin (BSA) was employed as a model protein to gain deeper insights into the release behavior of Pluronic-based hydrogels. Its water solubility and stability under various physiological conditions facilitated the incorporation of BSA into the hydrogel matrix during the cross-linking process. This choice allowed for a more comprehensive understanding of the hydrogel’s release kinetics.

This study aims to analyze protein release from diacrylated Pluronic F127–based hydrogels. Unlike previous studies primarily focused on structural or mechanical characterization, this work systematically addresses protein release behavior as a function of network composition and environmental conditions. To date, the release of proteins from acrylated Pluronic F127 systems has not been reported. Protein release was investigated under two physiologically relevant pH conditions (7.4 and 5.4), across a range of hydrogel polymer concentrations and different bovine serum albumin (BSA) loadings. Acrylation of Pluronic F127 overcomes the mechanical limitations of conventional Pluronic hydrogels, which are characterized by poor structural stability in aqueous media. In the context of controlled biomolecule delivery, this improvement is particularly relevant, as network stability and mesh size directly influence protein diffusion, protection, and sustained release. Furthermore, the experimental release data were fitted to a kinetic release model not previously described for diacrylated Pluronic-based systems, thereby establishing a solid experimental and modeling framework that enables the rational design and tuning of Pluronic-based hydrogels for controlled protein release.

## 2 Materials and Methods

### 2.1 Materials

Pluronic triblock copolymer F127 (F127), Bovine Serum Albumin (BSA), 2-Hydroxy-4′-(2-hydroxyethoxy)-2-methylpropiophenone (Irgacure 2959), acryloyl chloride, dichloromethane, triethylamine, ethyl ether, metallic iodine and potassium iodide were purchased from Sigma-Aldrich. Phosphate-Buffered Saline (PBS) was prepared using sodium chloride, disodium hydrogen phosphate, and potassium dihydrogen phosphate (Sigma-Aldrich), together with potassium chloride (PanReac AppliChem). Acetate buffer solutions were prepared from acetic acid and sodium acetate, both obtained from Sigma-Aldrich. For the Lowry protein assay, sodium hydroxide, sodium carbonate, sodium tartrate dibasic dihydrate, and Folin–Ciocalteu reagent were supplied by Sigma-Aldrich, while copper­(II) sulfate pentahydrate was purchased from PanReac AppliChem. All reagents were used as received, without further purification. Nitrogen was purchased from Air Liquide.

### 2.2 Methods

#### 2.2.1 Diacrylated Pluronic F127 Synthesis

Synthesis of diacrylated Pluronic F127 (F127DA) was described above for Cellesi et al.^46^ Briefly, commercial F127 was dissolved in 100 mL of CH_2_Cl_2_. Then, under a nitrogen atmosphere, a 10-fold molar excess of both triethylamine and acryloyl chloride is added dropwise. The reaction was stirred overnight in a nitrogen atmosphere. Following that, the solvent was evaporated using a rotary evaporator, and the obtained polymer was precipitated in cold ethyl ether and subsequently filtered in a Büchner funnel. The product is a white powder that was vacuum-dried for 2 days.

### 2.3 BSA Loading and Hydrogel Preparation

BSA was dissolved in pH 7.4 phosphate-buffered saline (PBS) at two concentrations (1 g/L and 20 g/L). Then, desired amount of F127DA polymer was added to the BSA solution with 0.1% wt of Irgacure 2959 photoinitiator. The solution was stirred and was left to stand overnight at 4 °C in the dark. Then, the cold solution was placed in cylindrical molds (10 mm diameter, 10 mm height) and was heated to the gel phase. Cross-linking was carried out via photoinitiated activation under ultraviolet light irradiation at 365 nm for 30 min. Hydrogels were stored at 4 °C for further characterization.

In this study, hydrogel samples based on diacrylated Pluronic F127 loaded with Bovine Serum Albumin (BSA) were prepared, varying the proportions of polymer and protein. The selected polymer concentrations for the hydrogels were 20, 25, and 30% w/v. The BSA solutions were prepared at two concentration levels: 1 g/L and 20 g/L.

An encapsulation efficiency (EE%) of 100% was assumed, since F127DA was added to the protein solution and cross-linked *in situ*. Entrapment content (EC%) for each sample was quantified as the proportion of the biomolecule within the matrix, expressed relative to the total of the system. This parameter was calculated according to eq 1 and is shown in Table 1 for each sample.

1 $$EC\;\left(\%\;w/w\right)=\frac{mass\;of\;entrapped\;BSA}{mass\;of\;polymer\;in\;the\;hydrogel}\times 100$$

**Table 1:** Nomenclature of the Hydrogels Used for the Different Polymer and BSA Concentrations

name	polymer concentration (% w/v)	Initial BSA concentration (g/L)	EC (%)
F127DA30/0.3	30	1	0.3
F127DA25/0.4	25	1	0.4
F127DA20/0.5	20	1	0.5
F127DA30/6.7	30	20	6.7
F127DA25/8	25	20	8
F127DA20/10	20	20	10

The nomenclature follows the format: F127 denotes the polymer used (in this case, Pluronic F127), the suffix “DA” indicates that it is the diacrylated polymer product, followed by the polymer concentration. For protein-loaded hydrogels the second number refers to the EC. For clarity. Table 1 shows the summary of the nomenclature used.

#### 2.3.1 Nuclear Magnetic Resonance (NMR)

Proton Nuclear Magnetic Resonance (^1^H–NMR) spectroscopy was employed to monitor the F127DA formation reaction. Spectra were acquired on a Bruker Ascend 500 MHz spectrometer, and chemical shifts were referenced to the residual proton signal of the deuterated solvent used, CDCl_3_, which appears at 7.26 ppm under standard conditions. The data obtained were analyzed using MestreNova software.

#### 2.3.2 Differential Scanning Calorimetry (DSC)

Differential scanning calorimetry (DSC) measurements were performed on a DSC Q1000 TA Instruments. An aluminum hermetic pan containing the sample (3–10 mg) heated from −10 to 60 °C and a cooled from 60 to −10 °C at a sweep rate of 5 °C/min in a nitrogen atmosphere. All DSC measurements were performed on hydrated samples, with the polymer dispersed in an aqueous medium, in order to preserve the native micellar state of the system. Thermograms were used to calculate the Krafft temperature (the minimum temperature at which surfactant molecules become soluble and can form micelles in an aqueous solution) based on the maximum of the endothermic and exothermic peaks. The maximum of the endothermic peak observed at the heating ramp was used to determine the Krafft temperature. This value thereby represents the temperature at which the micellization process of the amphiphilic polymeric chains reaches its maximum rate. Experiments were conducted in duplicate.

#### 2.3.3 Rheological Analysis

Sol–gel transition temperature was studied through viscosity changes using a rotational rheometer Anton Paar MCR302 coupled with a Peltier system for temperature control. Experiments were conducted using a CP60 cone–plate system (60 mm of diameter and 1° of angle) equipped with a water trap to prevent water evaporation. One mL of cold solution at different concentrations of F127DA was placed in the rheometer dish previously cooled to 0 °C and the cone–plate was lowered to a 30 mm gap. Tests were performed in rotational mode with a fixed shear rate of 10 s^–1^ and subjected to a temperature ramp from 0 to 40 °C with a rate of 1 °C/min. All measurements were performed in triplicate with fresh samples.

#### 2.3.4 Freeze-Drying

Some samples were subjected to a freeze-drying process carried out in a *LyoBeta 6PL* (Telstar Co. Ltd.) freeze-dryer. Samples were derived from cross-linked hydrogels formed and handled under hydrated conditions prior to dehydration. First, hydrogels were freezing for 6 h at −40 °C, followed by a primary drying step in which first a vacuum at 200 μbar was applied and then held for 60 h at 25 °C. Lastly, a secondary drying step was carried out for 10 h at 40 °C. The freeze-dried samples were stored at 4 °C for further swelling index study.

#### 2.3.5 Swelling Index

The swelling index was estimated by measuring the weight of the hydrogel before and after immersing them in aqueous media at different times. The freeze-dried hydrogels were placed in vials with 2 mL of PBS (pH = 7.4) or acetate buffer (pH = 5.4) located in a thermostatised bath at 37 °C. At different times the samples were taken, dried thoroughly, and weighed. The experiments were conducted in triplicate.

#### 2.3.6 Fourier Transform InfraRed Spectroscopy (FTIR)

Fourier transform infrared spectroscopy (FTIR) was used for the characterization of hydrogels with and without BSA. Spectra were obtained on a Spectrum Two FTIR Spectrometer (PerkinElmer, Inc.) equipped with a Universal Attenuated Total Reflectance (UATR) accessory. FTIR analyses were carried out directly on the hydrogels in their hydrated state, both in the absence and in the presence of BSA, to preserve the native chemical environment of the polymer network. All FTIR spectra were collected using 16 cm^–1^ resolution with 16 scans in the wavelength range of 4000 to 500 cm^–1^.

#### 2.3.7 Fluorescence Spectroscopy

Conformational changes in BSA after UV radiation exposure were evaluated by fluorescence spectroscopy, as this technique allows analysis of the local environment of tryptophan residues, whose emission is sensitive to modifications in the protein’s tertiary structure. An FP-8550 spectrofluorometer was used, exciting the sample at a wavelength of 280 nm, corresponding to the maximum observed in the UV absorption spectrum. The emission spectrum was recorded over the range of 200–800 nm, maintaining the temperature constant at 25 °C.

#### 2.3.8 Cryo-Scanning Electron Microscopy (Cryo-SEM)

The internal morphology of the hydrogels was analyzed by scanning electron microscopy, after cryogenic freezing of the hydrated hydrogels with liquid nitrogen (Cryo-SEM), using a Gemini SEM 500 from Zeiss coupled to a PP3010 Quorum device, which ensured reproducibility between independent samples and minimized the risk of freezing-induced artifacts. ImageJ software was used to analyze the mean pore size and pore distribution.

#### 2.3.9 UV–Vis Spectrophotometry

UV–vis spectrophotometry was used for two purposes in this work. The equipment used was JASCO V-730 Spectrophotometer. Critical Micelle Concentration (CMC) was analyzed using the Iodine UV-absorption spectra method with Iodine as hydrophobic probe.^47^ Pluronic F127 concentrations ranging from 0.001 g/L to 0.4 g/L were prepared in PBS solution. Measurements were performed at 366 nm and were carried out in triplicate. On the other hand, the quantitative determination of the albumin concentration in the release assays was performed by the Lowry method^48^ by measuring the absorbance at 750 nm. For this purpose, an initial calibration curve (Figure S1) was constructed, spanning concentrations from 0.0375 g/L to 0.2375 g/L. All measurements were performed in triplicate.

#### 2.3.10 Mechanical Test

Discovery DMA 850 (TA Instruments) was used to subject hydrogels, in their hydrated and photo-cross-linked state, to static and dynamic compression test. A cylindrical geometry (*d* = 10 mm, *h* = 8 mm) was used, the strain ramp was 1.5 mm·min^–1^ and the frequency was 1 Hz, for each of the tests.

#### 2.3.11 In Vitro Release Experiments: Procedure and Mathematical Modeling

The different hydrogels with cylindrical geometry (*d* = 10 mm, *h* = 10 mm) were placed in 5 mL of both Phosphate Buffered Saline (PBS, pH 7.4) and acetate buffer (pH 5.4) in hermetically sealed glass vials. All experiments were performed on photocroslinked hydrogels in a hydrated state, thereby preserving their native structural organization. Each vial was placed in a thermostatic bath at 37 °C to mimic body temperature and kept under agitation during the analysis. Two mL from the solution was drawn approximately every 3 days until 30 days where the trial was finished and replenished with 2 mL of fresh buffer. The experiments were carried out in triplicate.

pH 7.4 and pH 5.4 were selected as representative boundary conditions corresponding to physiological and acidic pathological environments, respectively. The use of pH 7.4 is widely accepted to simulate normal tissue and blood conditions, whereas acidic pH values in the range of approximately 5.4–6.0 are commonly employed to model pathological microenvironments such as tumor tissue, which exhibit extracellular acidification due to altered metabolism and lactic acid accumulation.^49,50^ The selection of these two pH values is consistent with established experimental practices in the evaluation of pH-responsive hydrogel systems for biomolecule delivery.^51,52^ The chosen pH conditions enable a clear and systematic assessment of the hydrogel’s sensitivity to acidification, providing a robust framework for interpreting pH-dependent swelling and protein release behavior.

The controlled release mechanism was previously demonstrated by our research group and was described by them.^53^ This model divides the process into three steps; however, in our experiments, hydrogel degradation either does not occur or is negligible. Therefore, the degradation process will not be considered, and the mechanism will be divided into two steps. A mathematical model was developed for each step. This model allows the experimental data to be fitted with great precision according to the release mechanisms previously proposed.

##### 2.3.11.1 Initial Burst Release

The first step involves the rapid release of proteins located on the hydrogel surface. The process is driven by the solubility of the protein in the surrounding medium, with the external mass coefficient (*k*_*ext*_) characterizing the diffusion dynamics. This stage was modeling using Boyd′s Thin Film Diffusion Model^54^

2 $$1-F=-k_{\mathrm{ext}}\cdot t$$

where *F* is the fractional attainment of equilibrium (defined as *M*_t_ */M*_*∞*_, where *M_t_* and *M*_*∞*_, denote the cumulative amounts of protein released at any time *t* and at infinity, respectively) and *k*_ext_ is the external mass transfer coefficient. Experimental results showed a linear trend up to 6 days. Consequently, this first pattern of the release profile was modeled using this diffusion equation.

##### 2.3.11.2 Diffusion Through the Polymer Matrix

After the surface protein is released, diffusion from within the hydrogel matrix becomes the dominant mechanism. The release follows Crank’s solution to Fick’s Second Law of Diffusion under spherical assumptions^55^

3 $$\frac{M_{t}}{M_{\infty }}=1-\frac{6}{\pi^{2}}\sum_{n=1}^{\infty }\frac{1}{n^{2}}\cdot \exp\left(-\frac{n^{2}\cdot \pi^{2}}{R_{0}^{2}}\cdot D\cdot t\right)$$

where *D* is the apparent diffusion coefficient and *R*_0_ is the characteristic length of the spherical hydrogel radius, given by

4 $$R_{0}={\left(\frac{3r_{c}^{2}h}{4}\right)}^{1/3}$$

*r*_c_ represents the cylindrical radius (5 mm) and *h* the cylindrical height (10 mm) of the hydrogels.

Model assumptions included

- Homogeneous protein distribution within the hydrogel at *t* = *t*_*f*_^ext^ = *t*_0_^int^.
- The protein concentration remained below its solubility limit throughout the experiment.
- No protein accumulation on the hydrogel surface, ensuring a constant release rate.
- Perfect sink conditions throughout the experiment.

Under the acidic conditions examined, hydrogels with lower albumin content exhibited a markedly different behavior compared to their counterparts. These hydrogels underwent substantial degradation, releasing nearly all their albumin content within the first 12 days of incubation. In these cases, the experimental data conformed well to a first-order release model, characterized by a rate of release proportional to the remaining albumin content. Where *k*_1_ denotes the first-order release rate constant.

5 $$\frac{M_{t}}{M_{\infty }}=1-e^{-k_{1}\cdot t}$$

The *Solver* tool included in the Excel software and the OriginPro software were used for the adjustment of the mathematical model.

## 3 Results and Discussion

The results section will consist of two parts. First, synthesis and characterization of diacrylated Pluronic F127 (F127DA)^46^ have been developed. Pluronic F127 hydrogels are reported to typically have low mechanical strength, adhesion, and self-healing capabilities. To improve the mechanical stability of F127-based hydrogels, acrylate functionalization for hydroxyl groups at the two ends (F127DA) has been proposed. It would act as a cross-linker and therefore would be able to produce covalent networks with plus mechanical strength. In this step, the mechanical and physico-thermal properties were studied to select the optimal concentrations for hydrogel formation.

In the second part BSA was loaded into the diacrylated gel, and the swelling index of the lyophilized hydrogels and the kinetic profile of BSA release in an aqueous medium were studied.

### 3.1 Pluronic F127 Diacrylated Hydrogels: Synthesis and Characterization

This section focuses on the evaluation of the synthesis and characterization of the diacrylated polymer (F127DA), prior to loading with BSA, as well as the formation of hydrogels through their micellar properties and determination of the sol–gel transition temperature, with the aim of selecting optimal concentrations to study the protein release behavior of Pluronic-based hydrogels.

#### 3.1.1 Synthesis Checking by Nuclear Magnetic Resonance (NMR)

The commercial Pluronic F127 polymer and the reaction product were analyzed using proton Nuclear Magnetic Resonance spectroscopy (^1^H NMR) to assess whether the acrylation reaction of the polymer (as explained in Section 2.2.1) was successfully performed. The ^1^H NMR spectra of raw and diacrylated Pluronic F127 are presented in Figure 1. Characteristic signals are labeled with letters. The signals corresponding to the polymeric protons are observed in both spectra. Specifically, signal A corresponds to the protons of the primary carbon, signal B to the proton of the −CH–O–, and signal C to the protons of the −CH_2_–O– in poly­(propylene oxide). Signals D and E correspond to the protons of the carbons in poly­(ethylene oxide). The expanded view highlights the signals of the acryl group. Thus, the feasibility of the reaction was demonstrated through NMR spectroscopy, as confirmed by the appearance of signals I, J, and J′ (see enlarged Figure 1) corresponding to the protons of the acryloyl groups and the adjacent proton (G), which decouples due to its proximity to an ester group.

**Figure 1:**
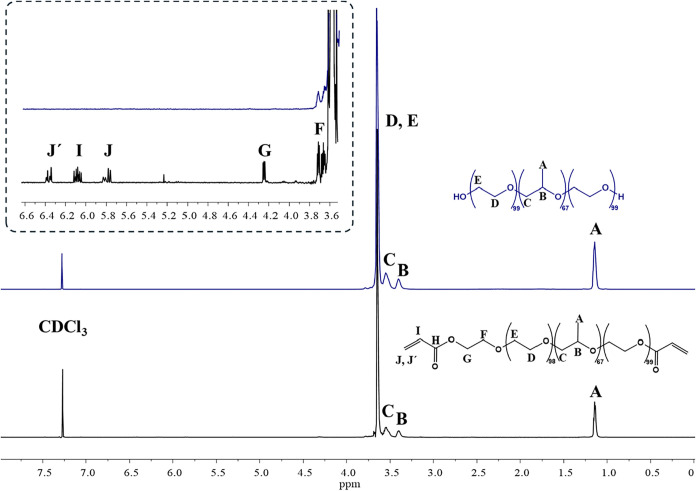
^1^H NMR spectra (500 MHz). ^1^H NMR resonance signals were measured relative to CDCl_3_ (7.27 ppm signal). The upper line (—) shows the ^1^H NMR spectrum of raw Pluronic F127 and the lower line (—) of diacrylated Pluronic F127.

**^1^H-NMR (500 MHz, CDCl_3_): δ (ppm):** 6.36 (m), 5.79 (m), 6.08 (m), 4.25 (t, *J* = 4.9 Hz), 3.71 (t, *J* = 4.9 Hz), 3.65 (s), 3.54 (s), 3.40 (s), 1.14 (s).

#### 3.1.2 Micelle Characterization of Diacrylated Pluronic F127

Figure 2 shows the critical micelle concentration (CMC) of F127DA using the Iodine UV_–_absorption spectra method. The CMC is defined as the concentration at which surfactant molecules begin to aggregate into spherical micelles.^56^ It is a critical parameter in the study of hydrogels, especially those based on amphiphilic polymers such as Pluronic, as micelle formation is strongly associated with the gelation behavior and the drug/protein loading efficiency of the material.^57^ The CMC was determined from the intersection of the curves defining the two trends shown by the absorbance versus the logarithm of F127DA concentration (Figure 2). For Pluronic F127 diacrylated, the CMC was found to be 0.176 ± 0.007 g/L (0.0176 ± 0.0007% w/v) at 298 K. In prior experiments, the CMC of the Pluronic F127 precursor was determined to be 0.145 g/L under comparable conditions. This slightly lower value suggests that the diacrylation process may subtly alter the hydrophilic–lipophilic balance of the polymer, resulting in a marginal increase in micelle formation threshold. The critical micelle concentration (CMC) reported in the literature for Pluronic F127 spans a wide range, from 0.126 g/L to 8 g/L.^58^ In the present study, both Pluronic F127 and Pluronic F127 diacrylate exhibited a value approaching the lower limit of this range.

**Figure 2:**
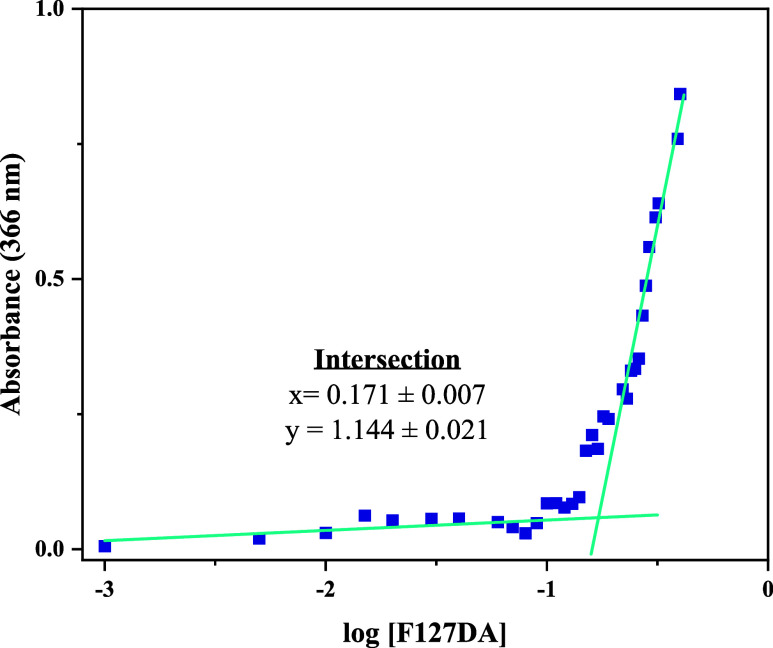
UV–vis spectrophotometry for critical micelle concentration at 298 K. F127DA concentration is expressed in g/L ranging from 0.001 g/L to 0.4 g/L.

The formation of micellar aggregates in Pluronic is also dependent on the temperature of the solution. Krafft temperature was studied for the potential hydrogel formation concentrations (ranging from 5% to 50% w/v) using DSC analysis. The analyses were conducted in a PBS solution, which minimally affects the association temperature due to the weak salting-out effect under these conditions. The DSC curves show a hysteresis process when the Pluronic solution was subjected to heating or cooling temperature ramps. Hysteresis lies in the kinetic effect of clustering and unclustering of Pluronic polymeric chains caused by the ramps of temperature transitions; in this work it was of 5 °C/min. Figure 3a shows heating and cooling thermograms. Krafft temperature decreased as the concentration of Pluronic in solution increased (Figure 3b), as expected. The proximity of the surfactant chains reduces solvation and enhances hydrophobic interactions between the polymer molecules. Consequently, the energy required for the polymer molecules to overcome repulsive forces and aggregate into micelles is reduced, thereby lowering the critical temperature for micelle formation.

**Figure 3:**
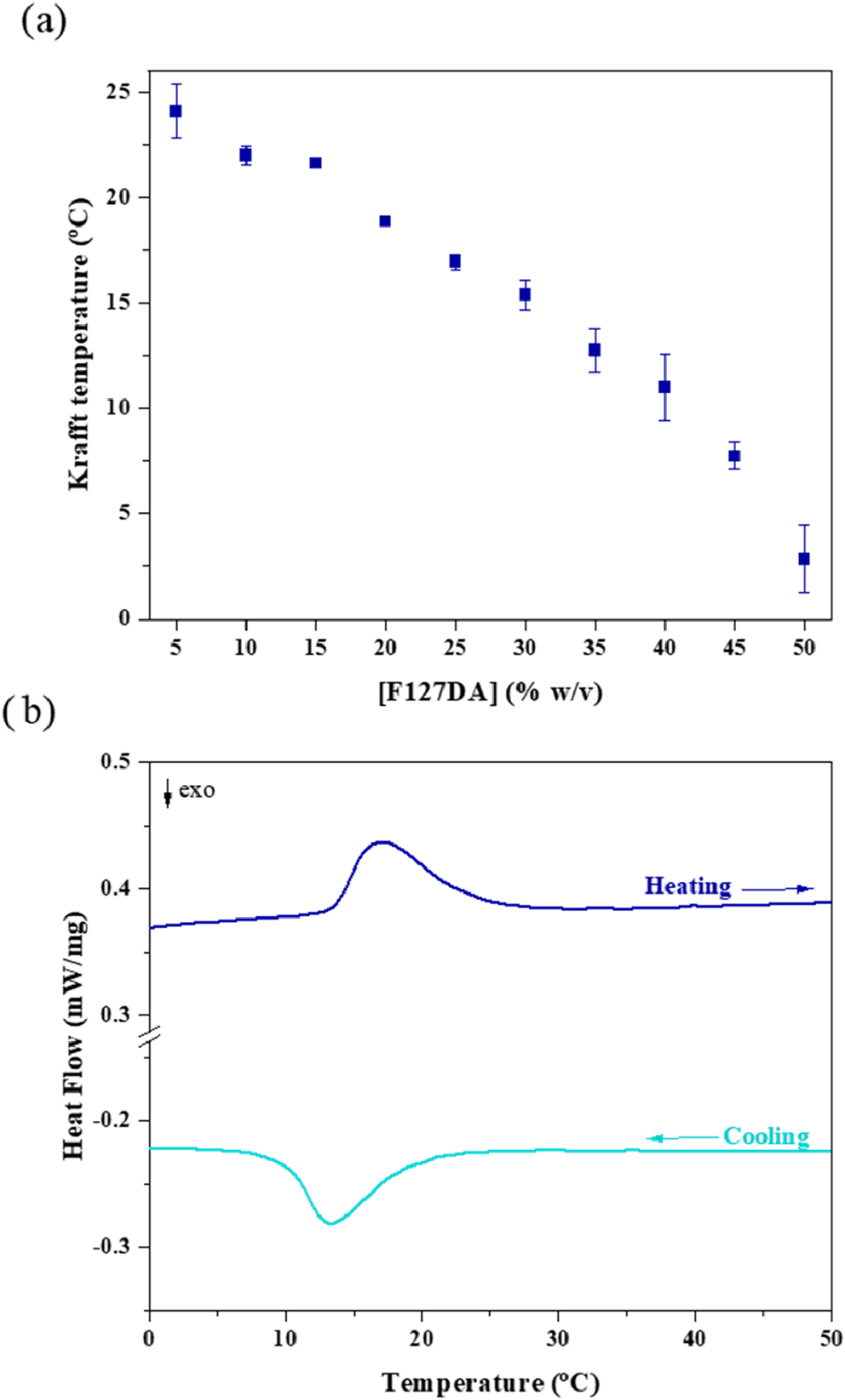
(a) Krafft temperature depending on F127DA concentration (5 to 50% w/v). Error bars are shown for duplicate tests. (b) DSC thermograms of F127DA with 25% w/v. The upper curve corresponds to the heating cycle and, the lower curve corresponds to the cooling cycle. Temperature ramp set at 5 °C/min.

#### 3.1.3 Sol–Gel TransitionTemperature

As described in Section 1, Pluronic F127-based hydrogels exhibit thermoreversible properties. At low temperatures, the solution is in a fluid state referred to as the *sol state*. When the temperature increases and the polymer chains aggregate into micelles, the solution transitions to a semisolid state, known as the *gel state*. The temperature at which this process occurs is referred to as the sol–gel transition temperature, typically an interval. This parameter is crucial for the stability, processability, and storage of hydrogels for their industrial application in controlled release or tissue engineering. Sol–gel transition temperature was studied through the rheological properties of the Pluronic solutions, particularly viscosity shown in Figure 4a. In Figure 4a, we can observe that at low temperatures, the solution exhibited low resistance to the stresses applied by the rheometer, indicating that it was in the sol state. The solution showed an abrupt increase in viscosity as it transitioned to the gel state. Finally, the viscosity of the solution stabilized with temperature variation, thus demonstrating the formation of the gel. The peak maximum of the first derivative of the viscosity curve was used to define the sol–gel transition temperature.

**Figure 4:**
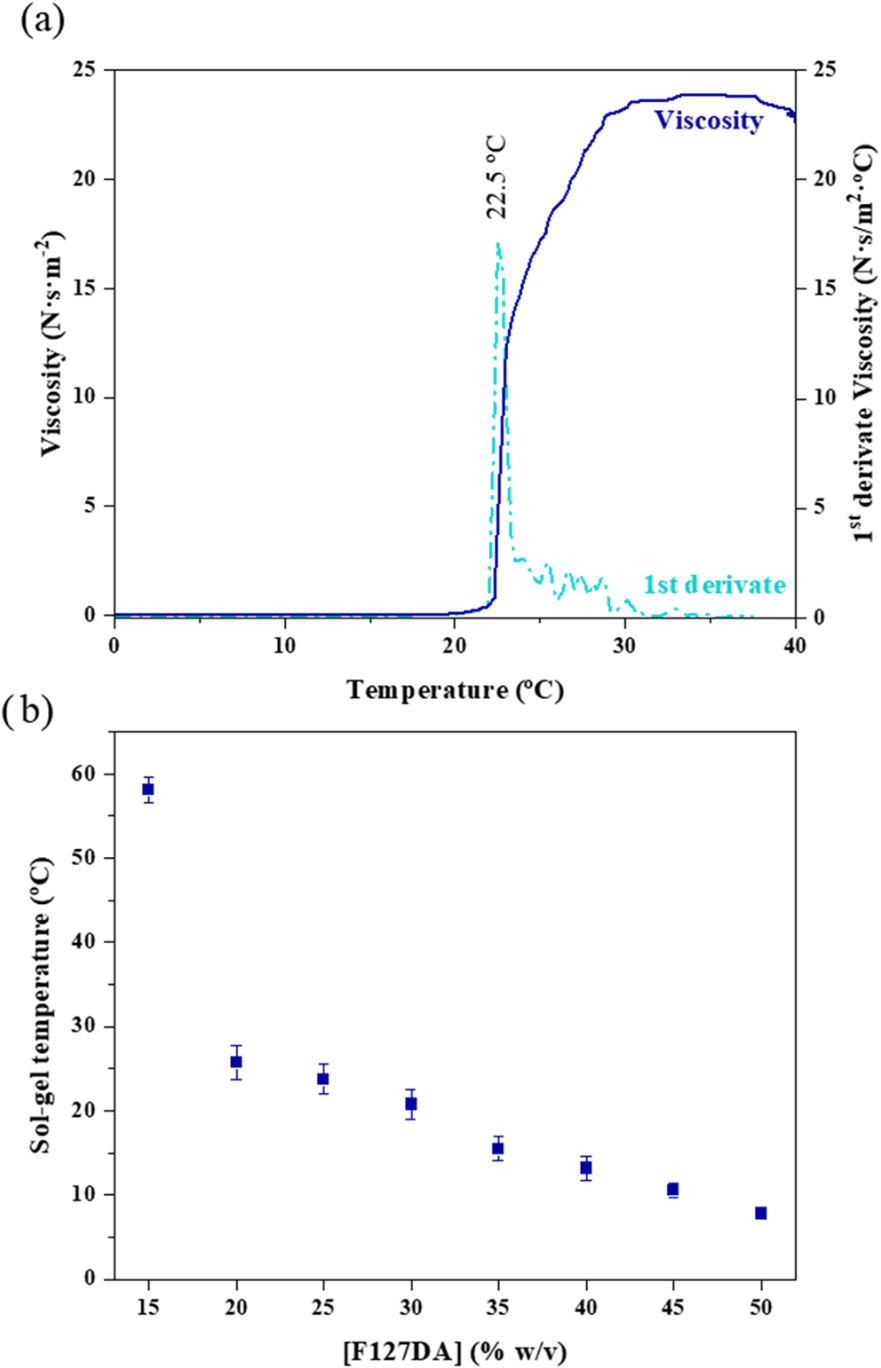
(a) Diagram of viscosity vs temperature for F127DA 25% w/v; sol–gel transition temperature was 22.5 °C. The first derivative of the viscosity curve is represented by the dashed line. (b) Sol–gel transition temperature depending on F127DA concentration (5 to 50% w/v). Error bars are shown for duplicate tests.

Analogous to the Krafft temperature, it was noted that the sol–gel transition temperature decreased as the concentration of F127DA increased (Figure 4b). Again, the highly concentrated domains of polymer chains at higher concentrations facilitate micellar aggregation, resulting in the sol–gel transition occurring at lower temperatures. It is noteworthy that Pluronic F127 concentrations below 15% did not exhibit a transition to the gel state. The 15% concentration showed an increase in viscosity, although the sample never reached a compact gel state. At higher concentrations, the sol–gel transition temperature demonstrated a linear dependence on concentration (Figure 4b).

In conclusion, the synthesis of the Pluronic F127 diacrylate compound was confirmed through NMR analysis. This section also evaluated the micellar properties of the F127DA polymer, focusing on critical micelle concentration and Krafft temperature. Understanding these properties is vital as they significantly influence the sol-to-gel transition, viscosity, drug loading, and material stability, all of which are crucial for biomedical applications. Additionally, viscosity was identified as a key factor for assessing the sol–gel transition, with F127DA-based hydrogels transitioning effectively to a gel state starting from a concentration of 20% w/v. Next sections will further explore concentrations above 20% w/v, specifically at 20%, 25%, and 30% in the presence of BSA.

#### 3.1.4 Mechanical Characterization

The photopolymerization of hydrogels under UV light serves two primary purposes: to transform thermoreversible hydrogels into thermally stable gels and, more critically, to enhance their mechanical strength. The incorporation of photoreactive terminal groups imparts significant advantages from both a research and application standpoint. From a research perspective, these hydrogels can be stored in their sol state, prior to curing, or in their gel state, offering flexibility in handling and experimentation. The UV-induced curing process enables precise structural stabilization, facilitating advanced characterization and tailored modifications. From an application standpoint, these materials provide exceptional adaptability, as they can be applied in sol or gel form to irregular defects in the human body and subsequently cured in situ, yielding enhanced mechanical and thermal stability. This dual-stage adaptability, combined with their biocompatibility and long-term stability, positions these hydrogels as ideal candidates for a broad range of biomedical applications, including tissue engineering and drug delivery systems.

The graphs obtained from the compression tests are shown in Figure 5, and the results are shown in Table 2. Compression tests revealed significant differences across the formulations. For F127DA20, the tan δ value, indicative of the damping ratio, was the highest at 0.64 ± 0.07, suggesting a more pronounced viscous behavior. Correspondingly, the storage modulus (G′) and Young’s modulus were the lowest, at 5.29 ± 0.34 kPa and 2.38 ± 0.56 kPa, respectively, indicating a softer, less rigid structure. As the concentration increased to 25%, the tan δ decreased to 0.33 ± 0.01, with an improvement in the storage modulus (93.48 ± 9.52 kPa) and Young’s modulus (11.74 ± 2.33 kPa), reflecting a shift toward a more elastic and stiffer matrix. At the highest concentration, F127DA30 exhibited the lowest tan δ value (0.16 ± 0.02), accompanied by the highest storage modulus (205.19 ± 20.63 kPa) and a remarkably improved Young’s modulus (131.86 ± 14.83 kPa), marking a substantial enhancement in rigidity and elastic behavior. This substantial increase in Young’s modulus indicates that concentration plays a pivotal role in enhancing the mechanical strength of the hydrogel, particularly at higher concentrations within the studied range. This trend suggests that increased polymer content leads to a denser and more interconnected network, resulting in improved structural integrity and mechanical robustness.

**Figure 5:**
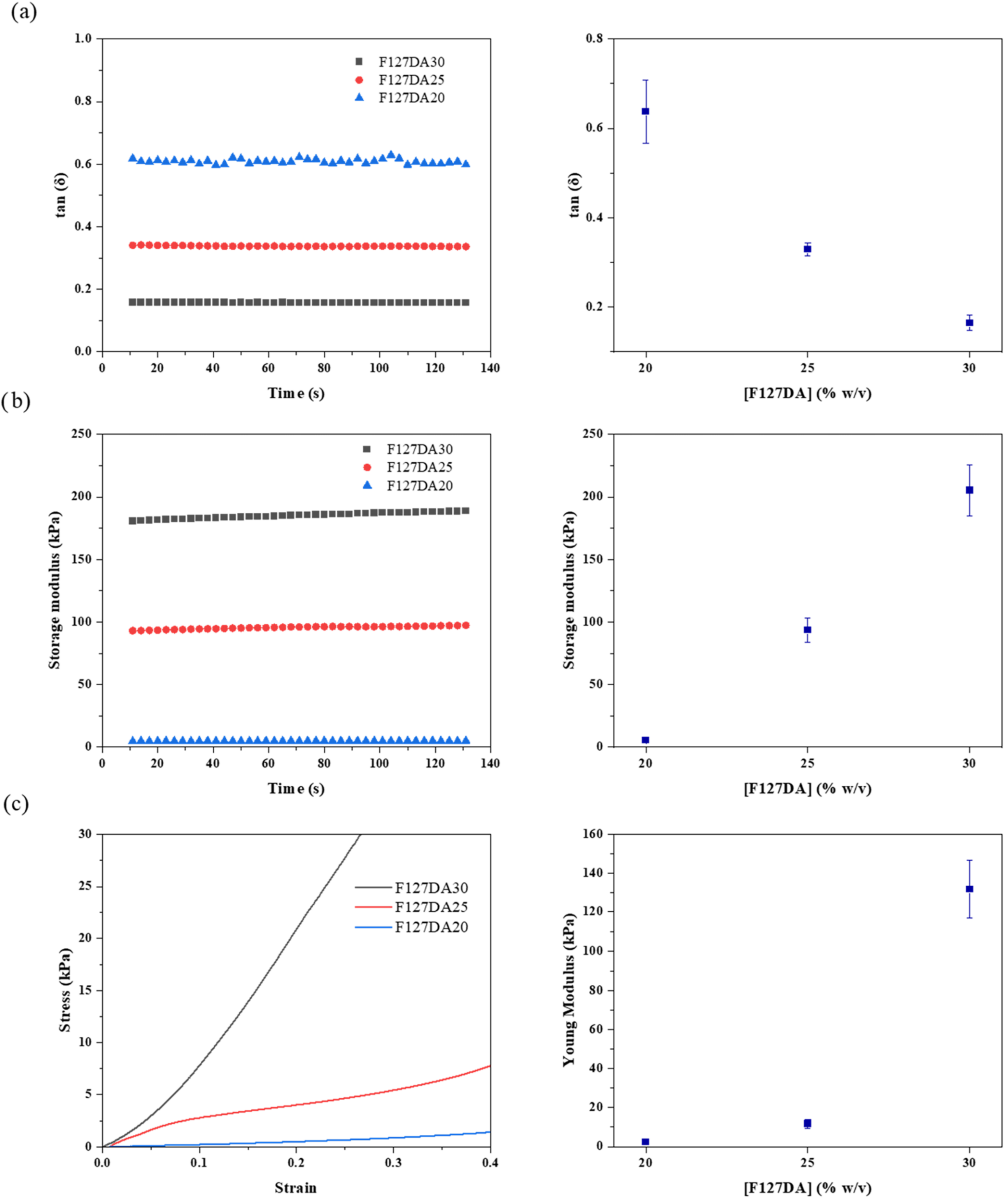
Comparative graphs of compression test results: (a) tan δ; (b) Storage modulus; (c) Young modulus.

**Table 2:** Mechanical Tests Results

	tan δ	storage modulus (kPa)	young′s modulus (kPa)
F127DA20	0.64 ± 0.07	5.29 ± 0.34	2.38 ± 0.56
F127DA25	0.33 ± 0.01	93.48 ± 9.52	11.74 ± 2.33
F127DA30	0.16 ± 0.02	205.19 ± 20.63	131.86 ± 14.83

Notably, uncured hydrogels could not be mechanically tested due to insufficient strength to demold, emphasizing that curing and increased cross-linking steps are essential to enhance the mechanical properties of these materials.

Strikingly, uncured hydrogels could not be mechanically tested due to insufficient strength to demold subsequent preparation and handling. Figure 6 shows that the uncured sample (on the left) lost its shape and collapsed after demolding, whereas the cured sample successfully maintained its structural integrity, emphasizing that curing and increased cross-linking steps are essential to enhance the mechanical properties of these materials.

**Figure 6:**
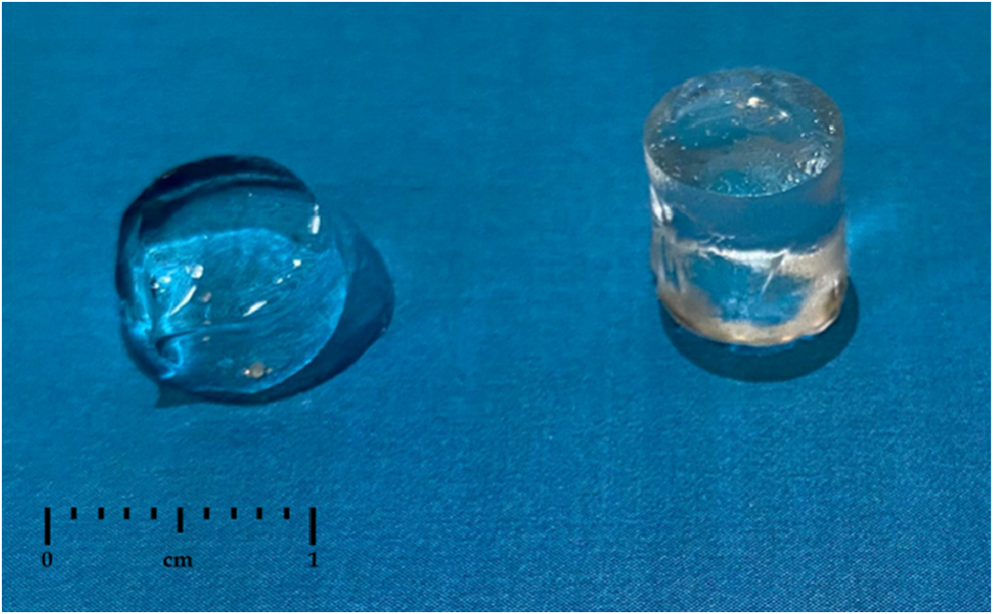
Two F127DA30 hydrogels, on the left hydrogel prior UV light and on the right hydrogel post-UV treatment.

#### 3.1.5 Morphological Characterization

The microstructure of the F127DA hydrogels at different concentrations was analyzed using scanning electron microscopy (SEM) (Figure 7). The pore morphology was predominantly spherical across all samples with no elongated or irregular pore geometries observed. The pore distribution remained homogeneous throughout the hydrogel volume at all concentrations, with no evidence of pore-free regions or distinct areas exhibiting visibly different pore sizes and followed a normal size distribution. The mean pore size did not exhibit significant differences, as the formation of the polymeric matrix in Pluronic-based hydrogels is driven by the self-assembly of micellar domains, which are influenced by the intrinsic properties of the polymer rather than its concentration. Importantly, the observed pore morphologies were highly reproducible across independently prepared samples and experimental conditions, providing an internal control against freezing-induced artifacts. Furthermore, the incorporation of BSA within the hydrogels did not lead to any noticeable alterations in their internal morphology, as can be seen in Figure 8 and demonstrated by the Duncan test (Table S5).

**Figure 7:**
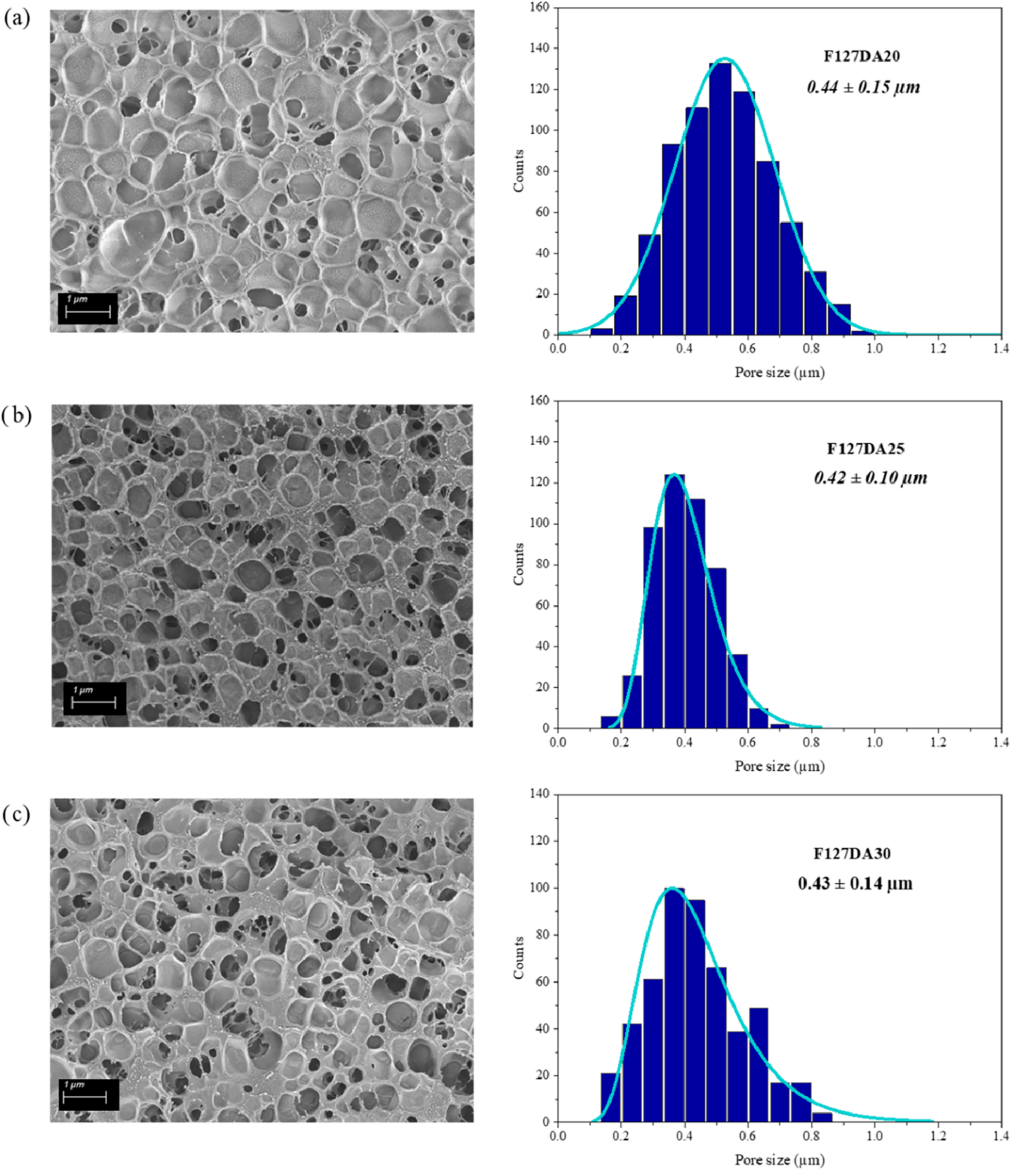
SEM images and pore size distribution of (a) F127DA20, (b) F127DA25 and (c) F127DA30 hydrogels.

**Figure 8:**
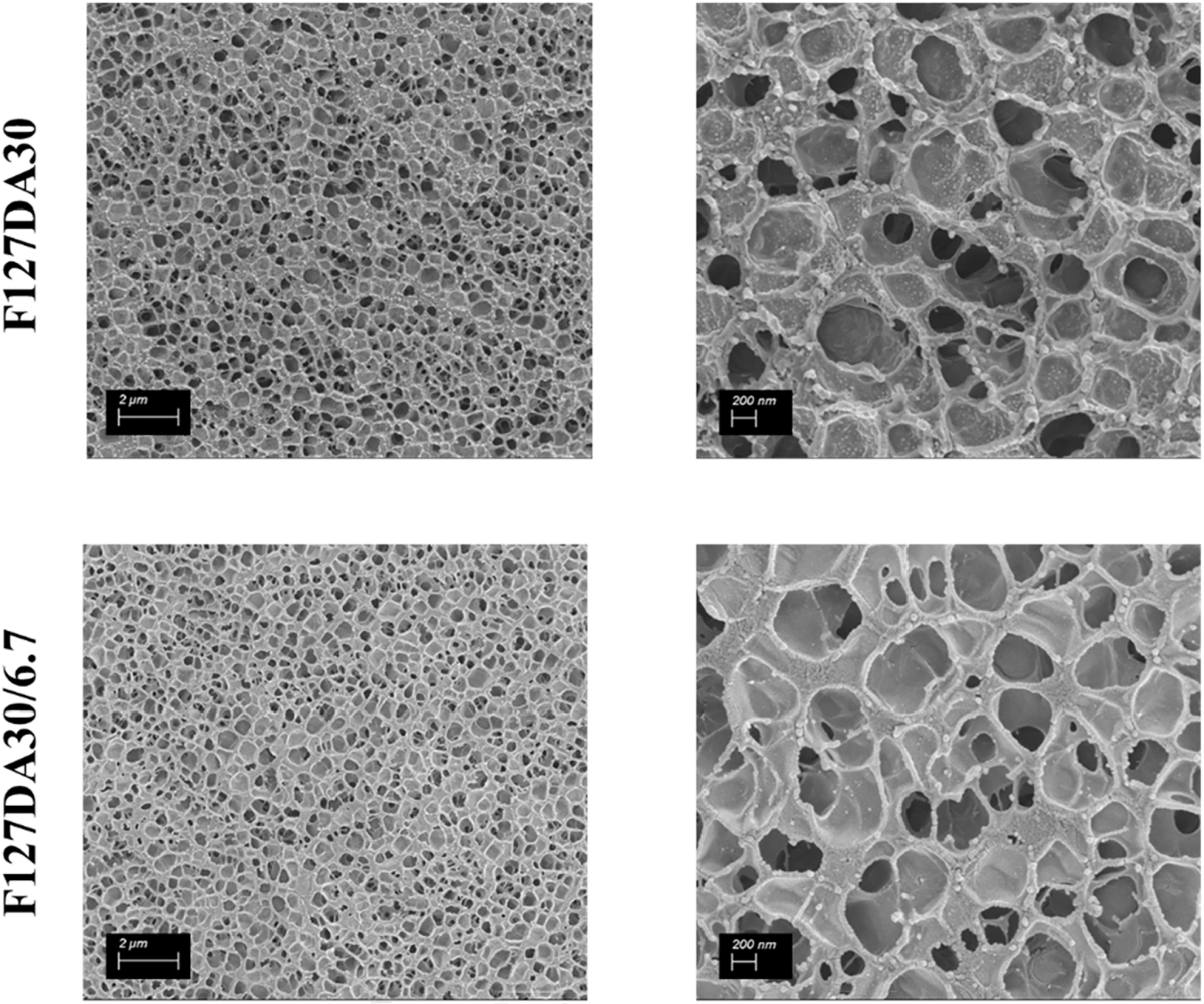
SEM images of F127DA hydrogels in the absence (F127DA30, top) and presence (F127DA30/6.7) of BSA. On the left, low magnification (2 μm); on the right, high magnification (200 nm).

### 3.2 Protein-Loaded Pluronic F127 Diacrylated Hydrogels: Synthesis, Characterization, and Functional Properties

Once the hydrogels for the study were selected, protein-loaded hydrogels were prepared for characterization and investigation of their properties. Freeze-drying was exclusively employed in the *Swelling index* section, as it was essential for the accurate assessment of this property. BSA was used as the model molecule for release assays due to its availability, stability, and well-characterized properties. During fabrication, the protein concentration within the hydrogels was set at two distinct levels: 1 g/L and 20 g/L BSA solutions. This approach was designed to cover a broad concentration range, enabling the assessment of release behavior under varying loading conditions. Such a strategy facilitates a more comprehensive understanding of the release dynamics governed by protein content.

Local acidification at wound sites has been repeatedly reported as a metabolic response associated with the healing process.^59–61^ This acidic microenvironment may significantly influence the behavior of hydrogels when applied in situ. Consequently, both the swelling and protein release dynamics of the hydrogels were evaluated under physiological (pH 7.4) and acidic (pH 5.4) conditions to explore the hydrogel’s behavior across different application scenarios. This comparison allows for a better understanding of the release mechanisms in both neutral and pathological environments.

#### 3.2.1 Characterization Protein-Loaded Hydrogels

##### 3.2.1.1 Fourier-Transform Infrared Spectroscopy (FTIR)

The FTIR spectra of the two hydrogels, F127DA25 with and without BSA, show several characteristic bands that reveal their molecular compositions (Figure 9, top). Both spectra show prominent bands at 1074 cm^–1^, corresponding to the C–O–C stretching vibrations of the Pluronic backbone. However, this band appears weaker when the hydrogel contains BSA and is slightly shifted to lower wavenumbers, likely due to the formation of molecular interactions, specifically hydrogen bonds between the amide groups of BSA and the carboxyl groups of Pluronic. Similarly, both spectra show bands at 2906 and 2978 cm^–1^, representing the C–H stretching of methylene and methyl groups, appearing more pronounced in the spectrum of the protein-containing hydrogel and can be corroborated in the BSA spectrum (Figure 9, bottom). The peak at 1640 cm^–1^, which appears in both spectra, can be attributed to the C=O stretching of the Pluronic, although it is more intense in hydrogel with BSA due to the additional contribution of the amide I band of the BSA. The spectrum containing BSA shows a band at 1530 cm^–1^, which is assigned to the amide II, which are absent in the pure hydrogel. Both can be observed in the BSA spectrum. Furthermore, the hydrogel with BSA shows a characteristic amide III band at 1248 cm^–1^, reflecting C–N stretching and N–H bending of the protein’s secondary structure. Moreover, the broad and steep band between 3700 and 3000 cm^–1^ appears similarly in both spectra and is associated with the O–H stretching of water. Overall, the inclusion of BSA introduces distinct vibrations associated with the protein, differentiating it from pure diacrylated Pluronic F127 hydrogel.

**Figure 9:**
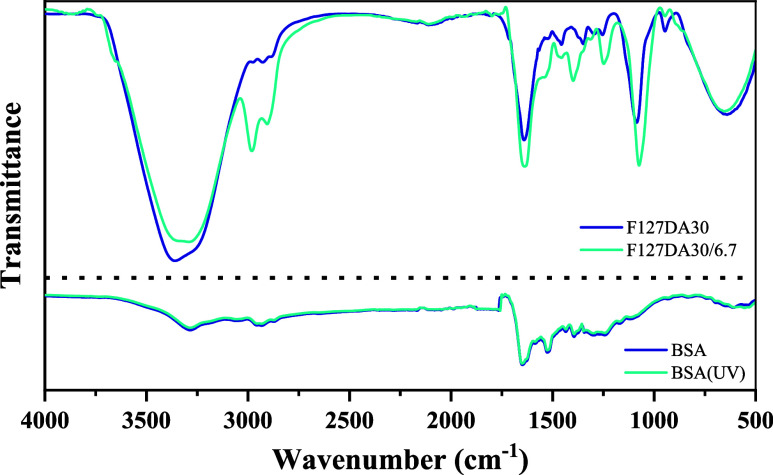
Up, FTIR spectra of F127DA30 hydrogels unloaded and loaded with BSA. Bottom, FTIR spectra of BSA and BSA exposed to UV radiation for 30 min.

FTIR analysis confirmed that UV radiation during the curing time of the hydrogels did not induce significant changes in the overall structure of the BSA protein, as both spectra exhibit nearly superimposable curves (Figure 9, bottom), suggesting similar molecular structures or environments.

The degradation of BSA local structure was further assessed by intrinsic protein fluorescence. UV irradiation is known to promote protein photo-oxidation through chromophore absorption (notably Trp/Tyr), with the overall extent of damage being governed by the irradiation dose (i.e., intensity and exposure time).^62,63^ Figure 10 presents the fluorescence emission spectra of native BSA and UV-irradiated BSA acquired under the same UV conditions applied to the hydrogels. Under these conditions, no shift in the emission maximum was detected relative to the native protein (λ_max_ = 339 ± 1 nm in both cases), and the spectral profile remained essentially unchanged. Alterations in the emission maximum or spectral shape are typically associated with conformational changes (typically red shifts); therefore, the spectrum indicates no significant UV-induced unfolding or aggregation at the applied dose. A small decrease in the emission intensity at λ_max_ (∼5%) was observed, which is consistent with a minor photochemical effect (e.g., oxidative modification or quenching) while remaining negligible in practical terms for the irradiation regime used in this study.

**Figure 10:**
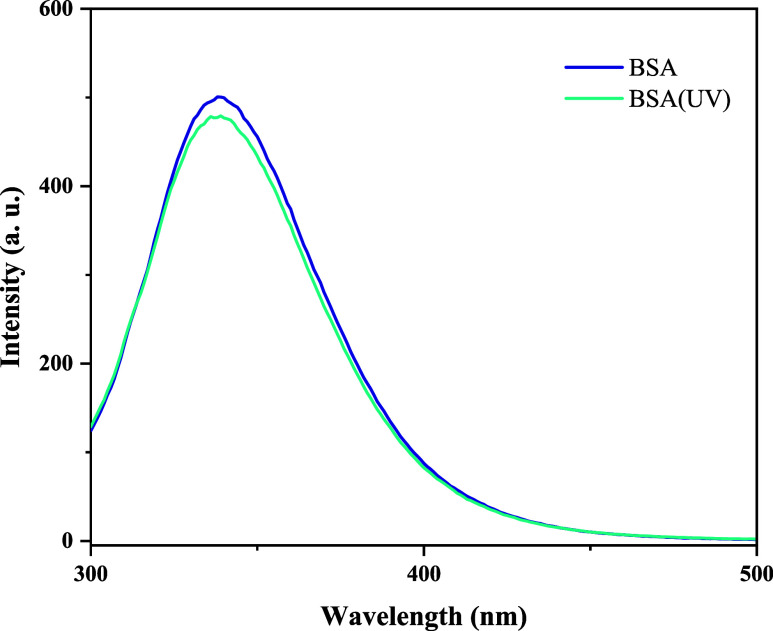
Fluorescence emission spectra for BSA and BSA exposed to UV radiation for 30 min. The spectra were obtained at 25 °C and λ_max_ = 280 nm.

##### 3.2.1.2 Swelling Index

The swelling index of a hydrogel quantifies its ability to absorb water and expand, commonly defined as the ratio between the weight of the swollen hydrogel and its initial dry weight. This parameter is critical for evaluating water retention, drug release efficiency, and responsiveness to environmental stimuli, providing valuable insights into the polymer network architecture and its functionality in biomedical applications such as tissue engineering and controlled drug delivery.^64,65^

To ensure consistency in the initial state of the hydrogels, the aqueous phase was first removed via lyophilization. Swelling experiments were then conducted using the freeze-dried samples. Hydration was assessed at 37 °C in an aqueous medium at pH 7.4 (PBS) and pH 5.4 (acetate buffer). Figure 11 depicts the time-dependent swelling index behavior of the F127DA30 samples. The swelling trend was consistent across all hydrogels. An initial increase in the swelling percentage was observed during the first hour, followed by a decrease. Subsequently, the swelling index increased again until reaching a stable equilibrium. Notably, the swelling behavior at pH 5.4 deviated from this pattern, with the hydrogels absorbing less aqueous solution and at a slower rate under acidic conditions.

**Figure 11:**
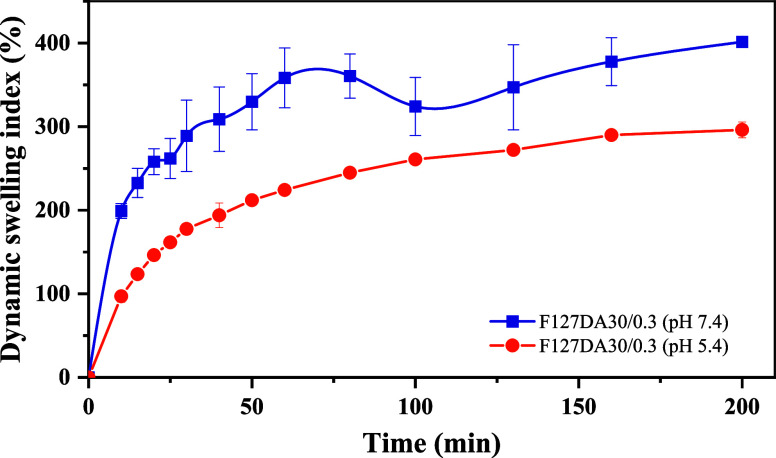
Dynamic swelling index (%) of F127DA30/0.3 hydrogels at different aqueous solutions, PBS (■, pH 7.4) and acetate buffer (■, pH 5.4). Error bars indicate standard deviations from triplicate measurements.

The observed swelling behavior at physiological pH is likely governed by the viscoelastic dynamics of the polymer network. Upon hydration, polymer chains expand and increase their conformational mobility, reaching a peak swelling state. However, over time, the system tends toward a lower-energy state, causing the polymer chains to relax and contract, expelling absorbed water molecules until a stable degree of hydration is reached (200 min). Conversely, under acidic pH conditions, the hydration rate is slower, preventing the development of an absorption inertia that would drive the system into a higher-energy state than its equilibrium hydration state. The reduced swelling observed at pH 5.4 can be attributed to the proximity of this value to the isoelectric point (pI 4.5–4.9) of bovine serum albumin (BSA). At this pH, BSA molecules exhibit minimal negative net charge, leading to decreased electrostatic repulsion and increased molecular aggregation within the hydrogel matrix. This results in a denser polymer network structure that restricts water uptake.^66^

Figure 12 shows the maximum swelling degree achieved for each of the prepared hydrogels. It can be observed that hydrogels with the lowest concentration of F127DA exhibited the highest degree of hydration. Conversely, in hydrogels with 25% and 30% w/v polymer content, the degree of hydration decreased. This phenomenon can be attributed to the increased cross-linking density and steric hindrance, which result in a more compact structure with reduced permeability to solvent infiltration into the polymeric matrix.

**Figure 12:**
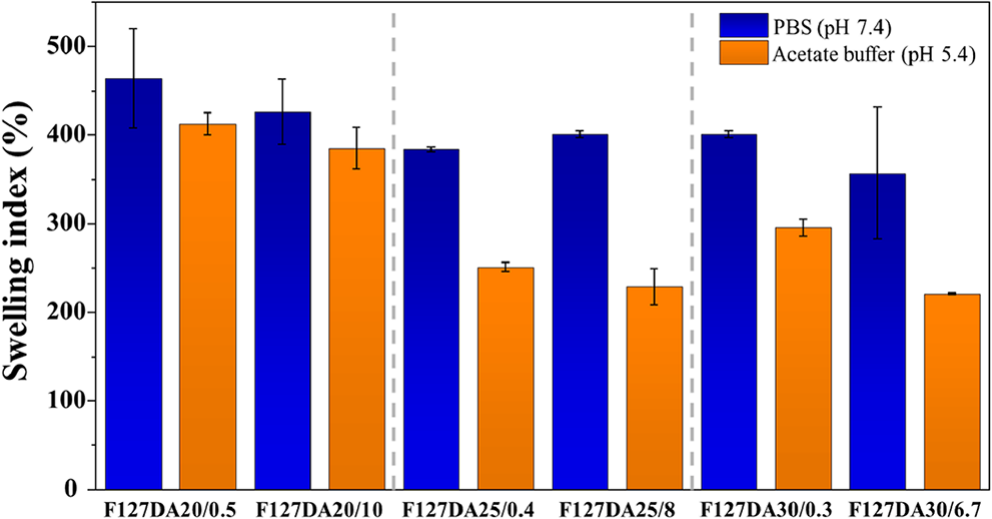
Maximum swelling index (%). Hydrogels were prepared with varying polymer concentrations (20, 25, and 30% w/v) and BSA concentrations (1 and 20 g/L). Error bars indicate standard deviations from triplicate test.

The pH significantly influenced the swelling capacity of the hydrogels, with a decrease under acidic conditions. At pH 5.4, hydrogels exhibited a lower degree of swelling compared to their behavior at physiological pH. This behavior is particularly relevant when considering the potential applications of hydrogels, as the healing process of injuries or wounds is often accompanied by local acidification.^67^

#### 3.2.2 In Vitro Release of BSA Kinetics Study

The protein release study was conducted over a period of 30 days, for physiological (7.4) and acidified (5.4) pH. Initially, each BSA concentration exhibited a distinct relative release profile (Figure 13). After 30 days, hydrogels with lower BSA content had released nearly their entire BSA load (left side of Figure 13). In contrast, hydrogels with higher BSA content showed a relatively lower release, with approximately 4–7% of the initial content being released (right side of Figure 13).

**Figure 13:**
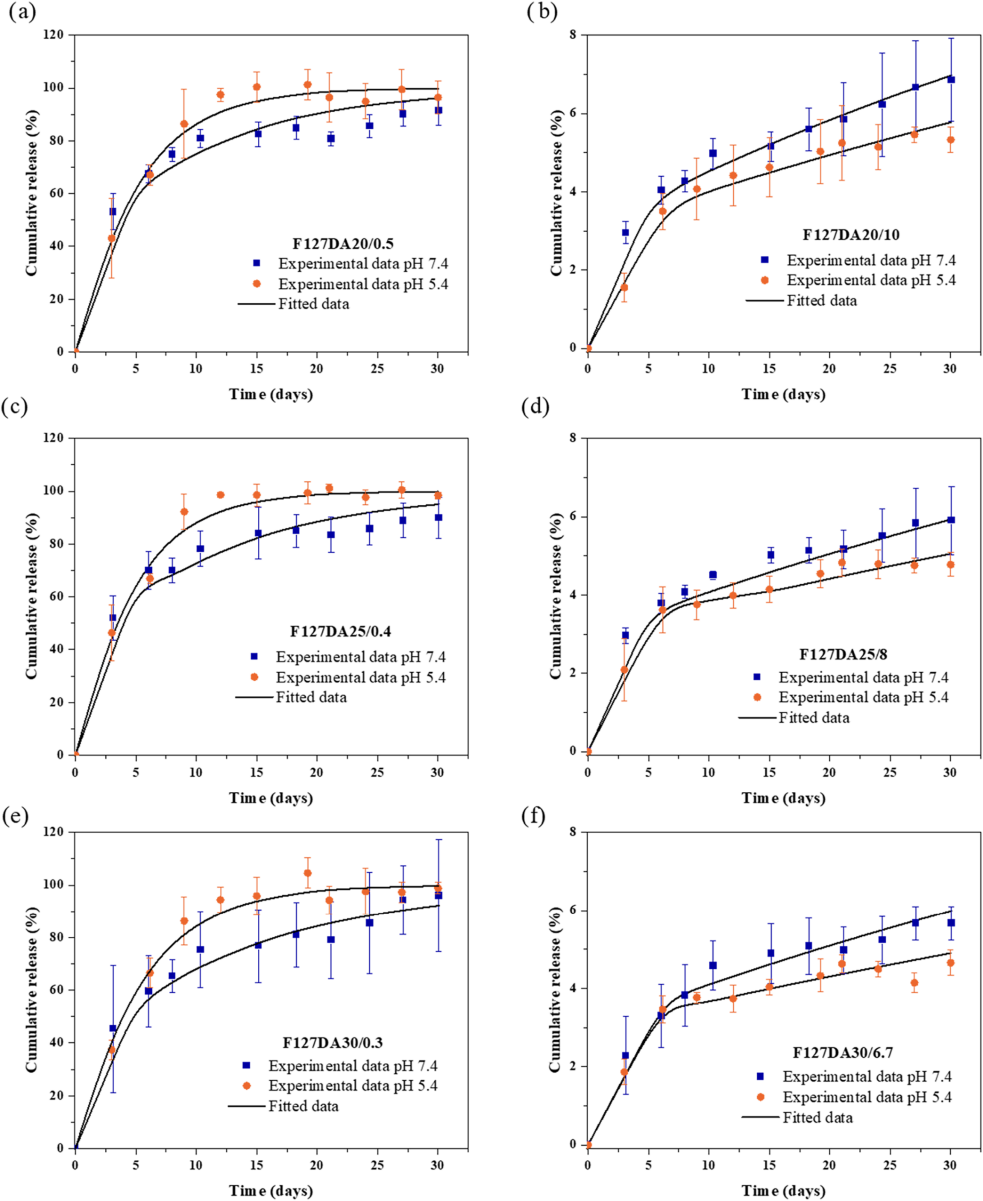
Cumulative release profiles of different hydrogel formulations based on Pluronic F127. The symbols show the experimental data, and the curves are the calculated theoretical values. Error bars are shown for triplicate tests. (a) F127DA20/0.5, (b) F127DA20/10, (c) F127DA25/0.4, (d) F127DA25/8, (e) F127DA30/0.3, (f) F127DA30/6.7.

Initially, the hypothesis that this behavior was due to a limitation in BSA solubility in the surrounding medium was rejected. This hypothesis was ruled out since the BSA concentration in the release experiments never exceeded 0.3 g/L at any time, significantly lower than the maximum solubility limit reported in the BSA technical data sheet (Sigma-Aldrich), which indicates solubility up to 40 g/L in water. Therefore, the working concentration remained well below the saturation limit, thereby maintaining sink conditions throughout the entire study.

The release profile was fitted to a two-step kinetic model, as detailed in Section 2.3.10, in which all the physicochemical interactions and diffusional resistances of any corresponding step are summarized by a single effective adjustable parameter. The model parameters *k*_ext*,i*_ and *D*_*i*_ were determined by minimizing the sum of squared deviations between predicted and experimental values. Figure 13 illustrates the model fit for all release profiles, where solid lines are the model predictions, and points represents experimental values.

It is noteworthy that hydrogels with low BSA content, when immersed in acetate buffer solution (pH 5.4), underwent degradation between days 9 and 12 of the release period, resulting in the near-complete release of the stored albumin within this time frame (left side of Figure 13). This behavior is readily rationalized by acid-catalyzed hydrolysis of ester linkages introduced during network formation, a well-established degradation route for ester-containing hydrogel networks and related polymer matrices, with degradation kinetics that are strongly pH-dependent.^68^ This observation constitutes an exception to the proposed release model. For these cases, the experimental data were fitted to a first-order kinetic release model, as detailed in Section 2.3.10. The finding suggests that BSA plays a role in stabilizing the hydrogel structure, as hydrogels with high BSA content did not exhibit such degradation under the same buffer conditions. This stabilizing effect may be attributed to physical interactions between BSA and Pluronic F127, including hydrogen bonding and electrostatic interactions. Additionally, at pH 5.4, which is close to the isoelectric point of BSA (∼4.7), BSA may act as a local buffer, mitigating solvent ingress through osmotic effects and thereby protecting the polymer network from acid-catalyzed hydrolytic degradation.^69^

The proposed two-step model demonstrates a satisfactory fit to the experimental data, supporting the hypothesis that the release process is governed by an initial stage dominated by external diffusion, followed by a second stage in which internal diffusion becomes the rate-limiting factor. This behavior is characteristic of solute diffusion within particles that are challenging to access due to the presence of diffusional resistances imposed by an intact, undegraded matrix, a phenomenon frequently observed in similar systems.^49,70^

The effect of varying the polymer/protein ratio has been studied and confirmed for three polymer concentrations. Table 3 summarizes the fitted values for the model parameters rearranged according to polymer/protein ratio. The results demonstrate that the variables *k*_ext_ and *D* are significantly influenced by the Pluronic F127/BSA ratio. Generally, as the Pluronic F127/BSA ratio decreases, both *k*_ext_ and *D* show a consistent increase. For instance, in formulations with a high proportion of BSA relative to F127DA (F127DA20/10, F127DA25/8, and F127DA30/6.7), the values of *k*_ext_ and *D* are notably lower (in the order of 10^–6^ s^–1^ and 10^–12^ m^2^/s, respectively), consistent with release kinetics dominated by diffusion limitations imposed by the polymeric matrix rather than by buffer exhaustion or loading effects. This behavior can be attributed to the higher density of the polymeric matrix and the associated diffusional resistances.

**Table 3:** Fitted Values for Parameters of the Proposed Model

				pH 7.4			pH 5.4		
	[Pluronic F127] (% w/v)	[BSA]_0_ (g/L)	EC (%)	*k*_ext_ (s^–1^)	*D* (m^2^/s)	*R* ^2^	*k*_ext_ (s^–1^)	*D* (m^2^/s)	*R* ^2^
F127DA20/10	20	20	10	2.01 × 10^–06^	1.30 × 10^–11^	0.9789	1.55 × 10^–06^	8.45 × 10^–12^	0.9867
F127DA25/8	25	20	8	1.93 × 10^–06^	9.00 × 10^–12^	0.9559	1.69 × 10^–06^	6.00 × 10^–12^	0.9829
F127DA30/6.7	30	20	6.7	1.62 × 10^–06^	9.20 × 10^–12^	0.9765	1.59 × 10^–06^	5.50 × 10^–12^	0.9730
F127DA20/0.5	20	1	0.5	3.43 × 10^–05^	8.50 × 10^–09^	0.9227	5.68 × 10^–05^ ^a^		0.9871
F127DA25/0.4	25	1	0.4	3.51 × 10^–05^	7.70 × 10^–09^	0.9515	6.09 × 10^–05^ ^a^		0.9863
F127DA30/0.3	30	1	3.3	3.00 × 10^–05^	6.30 × 10^–09^	0.9611	5.29 × 10^–05^ ^a^		0.9850

a The data refer to the first-order release rate constant, *k*_1_ (s^–1^).

Conversely, in formulations with a lower proportion of F127 relative to BSA (F127DA20/0.5, F127DA25/0.4, and F127DA30/0.3), the values of *k*_ext_ and *D* increase significantly, reaching the order of 10^–5^ s^–1^ and 10^–9^ m^2^/s, respectively. This increase suggests that at lower F127 concentrations, both external and internal resistances are reduced, facilitating a faster release of the solute.

Consequently, regarding controlled release products, formulations with higher F127 concentrations emerge as particularly promising candidates. This advantage, combined with their significantly improved mechanical properties, makes the higher-concentration hydrogels studied in this work highly attractive for potential industrial implementation.

Additionally, the trends indicate that *k*_ext_ is more sensitive to variations in the F127/BSA ratio than *D*, underscoring the importance of external diffusion mechanisms during the initial stages of the release process. It is also important to note that the decrease in *D* at higher F127 concentrations reflects a common phenomenon in polymeric systems: solute mobility within the matrix is restricted not only by the physical structure but also by specific interactions between the polymer and the solute, such as hydrophobic or van der Waals forces. This is particularly relevant for large molecules like BSA, which possess considerable size and multiple potential interaction sites.

These coefficients, which have not been determined until now, are essential for the design of release systems and underscore the need for diffusional data to optimize such systems for industrial applications.

#### 3.2.3 Comparative Performance and Potential Applications

Controlled release systems have become fundamental tools in tissue engineering and drug delivery, enabling sustained and targeted delivery of bioactive molecules. In this study, we demonstrate that diacrylated Pluronic F127 hydrogels provide a sustained release profile of Bovine Serum Albumin (BSA), with release kinetics that showed significant variability depending on both BSA content and pH of the release medium. In this context, we compare the performance of Pluronic-based hydrogels with other widely used systems.

To the best of our knowledge, studies employing photo-cross-linked diacrylated Pluronic F127 hydrogels for the investigation of BSA release have not been previously reported. Although Choi et al.^71^ explored a related diacrylated Pluronic F127 system, their work was conducted at very low polymer concentrations (1.4, 0.77, 0.50, 0.33, and 0.20 wt %), which resulted in the formation of nanocarriers rather than macroscopic hydrogel networks. Moreover, in that study the release behavior was modulated through temperature variations during the release process.

Several studies have investigated BSA-release behavior from alternative Pluronic F127–based hydrogel systems; however, it is noteworthy that all reported release experiments were conducted exclusively at physiological pH 7.4. In one representative approach, hybrid hydrogels composed of methacrylated bis-F127 covalently linked to thiolated hyaluronic acid were loaded with fluorescently labeled BSA and monitored over a 14-day period. These systems exhibited a pronounced initial burst release, with more than 60% of the cumulative protein released within the first 24 h, followed by a slower, progressive increase in release up to the end of the study.^72^ In another strategy, F127 was functionalized with α-lipoic acid to produce photo-cross-linkable hydrogels loaded with BSA, and the temperature dependence of release kinetics was examined at 25 and 37 °C. In this case, protein release was rapid and nearly complete at 25 °C, whereas a more sustained release profile was observed at 37 °C.^73^ Other works have focused on random copolymers combining Pluronic F127 with poly­(methyl vinyl ether-*co*-maleic anhydride), which significantly enhanced sustained BSA release when compared to unmodified F127 hydrogels, the latter showing very fast release (approximately 70% within 1 h and complete release within 24 h).^74^ Finally, injectable thermosensitive composite hydrogels based on Pluronic F127 and glycosaminoglycans, further modified with hyaluronic acid and loaded with BSA, have been reported to achieve cumulative releases of only 38–47% after 16 days.^75^ Collectively, these studies highlight the strong influence of chemical modification and formulation strategy on release behavior, while also revealing that systematic investigations beyond pH 7.4 remain largely unexplored.

F127DA hydrogels in this study exhibit several advantages over other widely studied delivery systems, making them highly suitable for sustained protein release in long-term tissue engineering and therapeutic delivery applications. Their thermoresponsive behavior allows for a sol-to-gel transition at physiological temperatures, enabling easy handling and potential injectable delivery. Furthermore, the polymer network formed after photo-cross-linking provides a stable structure that allows for controlled protein diffusion. It should be noted that the scope of the present study was primarily focused on the characterization of the hydrogel system and its protein release behavior. Additional aspects relevant to hydrogel-based drug delivery systems, such as polymer–protein interactions, the influence of ionic strength, and cytocompatibility, may be addressed in future studies.

In perspective, the release kinetics observed, together with the intrinsic versatility of the hydrogels, support a broad range of potential biomedical applications. For instance, the rapid degradation and release of low-concentration BSA hydrogels under acidic conditions suggest their suitability as topical dressings for mild to moderate wounds, potentially enabling the codelivery of therapeutics to promote tissue regeneration. Conversely, the controlled and sustained release exhibited by high-concentration formulations indicates promising utility in more demanding clinical contexts, such as in situ postsurgical applications, severe wounds, or even the targeted treatment.

## 4 Conclusions

This study has provided a thorough evaluation of the micellar, mechanical, morphological and thermo-physical properties of hydrogels based on Pluronic F127, terminally functionalized with photoreactive groups such as acrylates. Hydrogels with concentrations of F127DA above 20% w/v demonstrated stable gel formation at a sol–gel transition temperature below that of the human body, a finding of particular interest for potential in situ applications in tissue defects or wound healing. The morphology of the hydrogels remained consistent, as their internal structure is primarily governed by the micellar self-assembly of the polymer chains rather than polymer content. Mechanical characterization revealed significant improvements in rigidity and elasticity with increased polymer concentration, highlighting the role of cross-linking in enhancing structural stability.

Moreover, the release kinetics of the F127DA-BSA complex was governed by either a biphasic mechanism or conformed to first-order kinetics, depending on conditions. The protein release profile was significantly influenced by both the pH of the release medium and the initial BSA concentration within the hydrogel matrix. These variables modulated the diffusion and interaction dynamics of the protein within the polymeric network, allowing for fine control over release rates. These findings position F127DA hydrogels as versatile candidates for advanced drug delivery systems and scaffolds in tissue engineering, offering customizable properties for a wide range of biomedical and therapeutic applications.

## Supplementary Material



## Notes

Statistical Analysis (S1); Iodine UV-absorption spectra method (S2); Lowry method details and calibration curve (S3) (PDF)
