# Supplementary material for: Photo-Cross-Linked Pluronic F127 Hydrogels for Controlled Protein Delivery
Source: ACS Omega. 2026 Apr 29;11(18):26252–67. doi: 10.1021/acsomega.5c10776 (PMC13177001; doi:10.1021/acsomega.5c10776)
Supplement: Supplementary file 1 [file ao5c10776_si_001.pdf]

SUPPORTING INFORMATION

Photocrosslinked Pluronic F127 Hydrogels for Controlled Protein Delivery

Fernando Carrascosa, Ignacio Gracia, María Jesús Ramos, Jesús Manuel García-Vargas, Juan Francisco Rodríguez, María Teresa García*

Department of Chemical Engineering. University of Castilla-La Mancha. Facultad de Ciencias y Tecnologías Químicas. Avda. Camilo José Cela 12, 13071 Ciudad Real, Spain.

*Corresponding author:

e-mail: teresa.garcia@uclm.es

Phone: +34 926 05 28 51

S1. Statistical Analysis

To assess the statistical significance of the pore size results, an in-depth statistical analysis was conducted. The primary objective of this analysis was to determine whether pore size exhibited significant differences as a function of polymer concentration and upon the incorporation of BSA. Analysis of variance (ANOVA) was employed to evaluate overall differences among groups, followed by Duncan's multiple range test to perform pairwise comparisons of group means and to identify statistically significant differences between them. This statistical approach provides a robust framework for interpreting the experimental data and for drawing valid conclusions regarding the relationship between the studied variables. All statistical analyses were performed using Statgraphics Centurion software.

A descriptive statistical summary of the experimental data is first presented in *Table S1*. *Table S2* compiles the shape statistics and normality diagnostics of the pore size distributions corresponding to the different hydrogel formulations analysed. For all individual formulations, the standardised values of skewness and kurtosis fall within the commonly accepted range of −2 to +2, indicating a reasonable approximation to normality. This behaviour supports the assumption of normal data distribution and therefore justifies the application of parametric statistical analyses in the subsequent evaluation of the results.

Table S1. Statistical summary

	Average	*Standard deviation*	*Coefficient of variation (%)*	*Min*	*Max*
F127DA20	0.443	0.117	26.5	0.142	0.738
F127DA25	0.422	0.102	24.2	0.176	0.722
F127DA30	0.426	0.144	33.9	0.127	0.794
F127DA30/6.7	0.442	0.149	33.7	0.13	0.813
Total	0.434	0.128	29.5	0.127	0.813

Table S2. Shape statistics and normality diagnostics

	Range	*Standardised Skewness*	*Standardised Kurtosis*
F127DA20	0.596	-0.208	-1.931
F127DA25	0.546	1.848	-0.621
F127DA30	0.667	1.722	-1.572
F127DA30/6.7	0.683	1.578	-1.799

The ANOVA results (*Table S3*) indicate that the p-value associated with the F-ratio is greater than 0.05; therefore, no statistically significant differences were detected among the means of the four variables at a 95.0% confidence level. However, ANOVA assesses overall differences across all datasets simultaneously and does not provide information on pairwise comparisons. To evaluate potential differences between individual datasets, Duncan’s multiple range test was subsequently applied, with the results reported in Tables S4 and S5.

Table S3. One-way ANOVA table

Source of variation	*Sum of Squares*	*Degrees of Freedom*	*Mean Squares*	*F-Value*	*p-Value*
Between Groups	0.084	3	0.028	1.71	0.163
Within Groups	15.890	970	0.016		
Total (Corr.)	15.974	973			

Table S4 presents the paired-group comparisons for the different hydrogel formulations. An asterisk (*) in row 2 would indicate that the corresponding hydrogel pore size data exhibit statistically significant differences. However, no pairwise comparisons showed statistically significant differences at a 95.0% confidence level. Table S5 summarises the homogeneous groups based on statistical significance. In this analysis, no statistically significant differences are observed between levels that share the same number of asterisks (*). The discrimination between group means was performed using Fisher's least significant difference (LSD) procedure. Accordingly, Table S5 indicates that all the studied groups belong to a single homogeneous group in terms of statistical significance.

Table S4. Duncan´s multiple range test

Contrast	Sig.	Difference	+/- Límits
F127DA20 - F127DA25		0.021	0.022
F127DA20 - F127DA30		0.017	0.023
F127DA20 - F127DA30/6.7		0.002	0.022
F127DA25 - F127DA30		-0.004	0.024
F127DA25 - F127DA30/6.7		-0.019	0.023
F127DA30 - F127DA30/6.7		-0.015	0.024

Table S5. Duncan´s multiple range test

Level	n	Average	Homogeneous Groups
F127DA25	246	0.422	*
F127DA30	210	0.426	*
F127DA30/6.7	225	0.441	*
F127DA20	293	0.443	*

S2. Iodine UV-absorption spectra method

For the iodine UV-absorption method, with molecular iodine serving as a hydrophobic probe, a KI/I_2 stock solution was prepared by dissolving 0.5 g of iodine (I_2) and 1 g of potassium iodide (KI) in 50 mL of Milli-Q water and stored protected from light. A 1 g/L mother solution was prepared, from which working solutions ranging from 0.001 to 0.4 g/L were obtained. Subsequently, 25 µL of the KI/I_2 solution were added to 5 mL of each sample, and the vials were wrapped in aluminium foil to prevent photodegradation. The mixtures were incubated for 24 h at room temperature, after which their absorbance at 366 nm was recorded using a V-750 UV-Vis spectrophotometer. All measurements were performed in triplicate, and mean absorbance values were used for analysis.

S3. Lowry method details and calibration curve

First, a stock solution of bovine serum albumin (BSA) was prepared at 0.25 g/L in PBS and subsequently used to generate a series of calibration standards. Each standard was brought to an initial working volume of 1 mL with PBS. Based on these preparations, an initial calibration curve was established covering concentrations from 0.0375 g/L to 0.2375 g/L. The alkaline copper reagent was freshly prepared immediately prior to use

by combining sodium carbonate in 0.1 N NaOH, 1% (w/w) copper (II) sulfate, and 2% (w/w) sodium tartrate in a 100:1:1 ratio, following established Lowry assay formulations. For each assay tube, 5 mL of this reagent was added, gently mixed, and allowed to react for 10 min at ambient temperature. Subsequently, 0.5 mL of Folin–Ciocalteu reagent, previously diluted 1:1 with Milli–Q water, was introduced, followed by immediate agitation and a 30 min incubation to allow full colour development. Absorbance was measured at 750 nm using a V-750 UV-Vis spectrophotometer, and the resulting values were used to construct the calibration curve (*Figure S1***Error! Reference source not found.**). The same Lowry protocol was applied to all test samples, and their protein concentrations were determined by interpolating the corresponding absorbance values against the previously established calibration curve.

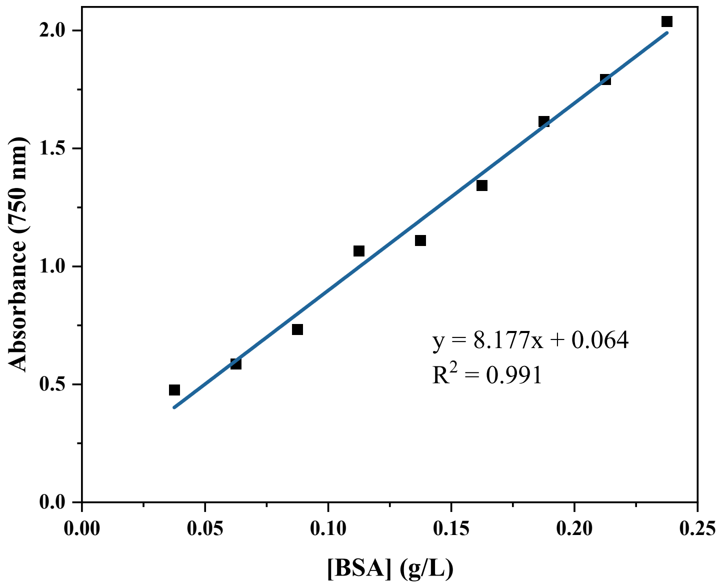

Figure S1. Experimental calibration curve for BSA absorbance.
